# A global transcriptional view of apoptosis in human T-cell activation

**DOI:** 10.1186/1755-8794-1-53

**Published:** 2008-10-23

**Authors:** Min Wang, Dirk Windgassen, Eleftherios T Papoutsakis

**Affiliations:** 1 Interdepartmental Biological Sciences Program, Northwestern University, Evanston, IL, USA; 2 Immunotherapy Development, Dendreon Corporation, Seattle, WA, USA; 3 Department of Chemical and Biological Engineering, Northwestern University, Evanston, IL, USA; 4 Department of Chemical Engineering and the Delaware Biotechnology Institute, University of Delaware, Newark, DE, USA

## Abstract

**Background:**

T-cell activation is an essential step of immune response. The process of proper T-cell activation is strictly monitored and regulated by apoptosis signaling. Yet, regulation of apoptosis, an integral and crucial facet during the process of T-cell activation, is not well understood.

**Methods:**

In this study, a Gene-Ontology driven global gene expression analysis coupled with protein abundance and activity assays identified genes and pathways associated with regulation of apoptosis in primary human CD3+ T cells and separately CD4+ and CD8+ T cells.

**Results:**

We identified significantly regulated apoptotic genes in several protein families, such as BCL2 proteins, CASPASE proteins, and TNF receptors, and detailed their transcriptional kinetics during the T-cell activation process. Transcriptional patterns of a few select genes (BCL2A1, BBC3 and CASP3) were validated at the protein level. Many of these apoptotic genes are involved in NF-κB signaling pathway, including TNFRSF10A, TNFRSF10B, TRAF4, TRAF1, TRAF3, and TRAF6. Upregulation of NF-κB and IκB family genes (REL, RELA, and RELB, NFKBIA, NFKBIE and NFKB1) at 48 to 96 hours, supported by the increase of phosphorylated RELA (p65), suggests that the involvement of the NF-κB complex in the process of T-cell proliferation is not only regulated at the protein level but also at the transcriptional level. Examination of genes involved in MAP kinase signalling pathway, important in apoptosis, suggests an induction of p38 and ERK1 cascades in T-cell proliferation (at 48 to 96 hours), which was explored using phosphorylation assays for p38 (MAPK14) and ERK1 (MAPK3). An immediate and short-lived increase of AP-1 activity measured by DNA-binding activity suggests a rapid and transient activation of p38 and/or JNK cascades upon T-cell activation.

**Conclusion:**

This comparative genome-scale, transcriptional analysis of T-cell activation in the CD4+ and CD8+ subsets and the mixed CD3+ population identified many apoptosis genes not previously identified in the context of T-cell activation. Furthermore, it provided a comprehensive temporal analysis of the transcriptional program of apoptosis associated with T-cell activation.

## Background

The adaptive immune response starts with the activation of the naive CD4+ and CD8+ T cells in the peripheral immune system. Successful T-cell activation requires the T-cell receptor complex (TCR) and the co-receptor CD28 [1], the ligation of which leads to several downstream signalling events, including activation of protein kinases such as LCK and ZAP70, activation of MAP kinase cascades, and activation and nuclear localization of crucial transcription factors including AP-1, NFAT, and NF-κB [2]. In contrast, TCR signaling alone without CD28 co-stimulation results in anergy and eventual cell death [3].

Apoptosis has been extensively examined in T cells post activation, such as activation-induced cell death (AICD), due to its essential role in eliminating unwanted lymphocytes and maintaining the homeostasis after fighting infection and inflammation [4]. However, the regulation of apoptosis and the balance between the anti-apoptotic and pro-apoptotic signalling (which is an essential part of the surveillance machinery) during the process of T-cell activation have not been examined.

Genome-scale transcriptional analysis is a powerful tool for understanding complex processes such as T-cell activation [5,6]. In a previous effort, using ontological analysis coupled with a comparative analysis of primary human T-cell activation in the CD3+ T cells and the two subsets, CD4+ and CD8+ T cells, we probed the common and potentially subset-specific immune response-associated transcriptome in T-cell activation [7]. In this study we focus on the differentially expressed genes associated with regulation of apoptosis, as well as essential apoptotic signalling pathways: the NF-κB signalling pathway, and MAP kinase signalling. We identified several potentially important apoptotic genes based on their patterns of expression and examined the protein expression of a select set of genes, most of which have not been previously discussed in T-cell activation.

## Methods

### Cells and culture system

CD3+, CD4+ and CD8+ T-cell cultures were set up as previously described [7]. Briefly, negatively-selected T cells (CD3+, CD4+, and CD8+) were activated with anti-CD3/anti-CD28 Mab conjugated to magnetic beads. Cell counting and sampling for flow cytometry and microarray analysis were carried out at 0, 4, 10, 48 and 96 hours in the CD3+ T-cell experiments, E1-E5, with cells from 5 independent healthy donors, and at 0, 6, 12, 24, 48 and 72 hours in the CD4+ T-cell and CD8+ T-cell experiments, E7-E11, with cells from 5 independent healthy donors. This study was approved by the Northwestern University IRB.

### Flow cytometry

The following monoclonal antibodies (Mabs) for flow cytometry were purchased from BD Biosciences (San Jose, CA) unless otherwise stated and included CD3 (FITC+PE), active CASP3 PE, phospho-NF-κB-p65 PE, phospho-p38 (MAPK14) PE, phospho-ERK1 (MAPK3) PE, PUMA (BBC3) (Cell Signaling Technology, Danvers, MA), BCL2A1 (Abcam, Cambridge, MA) and goat anti rabbit IgG PE (Jackson ImmunoResearch Laboratories, West Grove, PA). Flow cytometry was carried out as described[8,9]. Briefly, all samples were gated on forward scatter and on propidium iodide negative (PI-) to eliminate debris and dead cells. For intracellular detections, cells were first stained with anti-CD3-FITC and then fixed, permeabilized, and stained as previously described [10]. Quantibrite beads (BD Biosciences Immunocytometry Systems) labelled with different amounts of PE molecules were used to quantify surface or intracellular protein levels and normalize measurements between timepoints.

### Microarray experiments and data analysis

Total RNA was extracted, RNA integrity was evaluated and microarray experiments and data analysis were carried out as previously described [7]. Briefly, microarray data were normalized and further analyzed (identification of significant genes, hierarchical clustering, and Gene Ontology assignment) with 'MultiExperiment Viewer (MeV)' from The Institute for Genomic Research (TIGR) [11]. Raw and normalized data were deposited in the Gene Expression Omnibus (GSE6607 (CD3+ T-cell experiment), GSE7571 (CD4+ T-cell experiment) and GSE7572 (CD8+ T-cell experiment)) [12]. Within each population (three biological replicates using cells from three different donors), multi-class SAM (Significance Analysis of Microarrays) with a false discovery rate of < 1% was used to select genes that show statistically different expression between groups. A SAM group is defined here as all the samples belonging to the same timepoint regardless of donor. Briefly, there were 5 groups (0 hour, 4, 10, 48 and 96 hours) in the set of CD3+ experiments and 6 groups (0 hour, 6, 12, 24, 48 and 72 hours) in the set of CD4+ experiments and CD8+ experiments. Gene expression at each time point was compared to that of 0 hour in each experiment. Gene Ontology annotations, as curated by European Bioinformatics Institute, were retrieved from the Gene Ontology Consortium website [13]. The EASE (Expression Analysis Systematic Explorer) score in ontological analysis is a modified Fisher Exact Probability p-value [14] indicating the probability of finding by chance the same degree of enrichment on a Gene Ontology term in a set of genes. The lower the EASE score, the more significant is the enrichment, i.e. the less likely that degree of enrichment can be found by chance. Hierarchical clustering analysis was performed with the Euclidean distance metric. The list of genes associated with NF-κB signaling pathway was curated based on the information of Gene Ontology Consortium [13] per 'positive regulation of I-kappaB kinase/NF-kappaB cascade' and superarray [15] per 'NF-κB Signalling Pathway'. Information of superarray enriched our list with members of the Rel, NF-κB, and IκB families. The list of NF-κB target genes was curated based on the information of website ([16]), a collective information source of NF-κB research based on updated publications. The gene list of MAP kinase signalling pathway was curated and sorted based on information of Kegg website [17] per 'MAPK signalling pathway', superarray [15] per 'MAP Kinase Signalling Pathway', and NCBI website [18].

### AP-1 activity assay

DNA-binding activity of AP-1 was assessed using the TransBinding AP-1 ELISA kit (Panomics; Fremont, CA) as described [19]. Briefly, nuclear extracts were incubated with biotinylated AP-1-consensus-binding-sequence oligonucleotides and complexes were detected using a primary AP-1 antibody and a secondary antibody conjugated to horseradish peroxidase. This assay is analogous to the traditional electrophoretic mobility shift assay in that it measures the ability of a transcription factor from nuclear lysates to bind to a consensus-binding sequence of that transcription factor, and has been extensively validated [20,21].

## Results

### Anti- and pro-apoptotic genes in T-cell activation

As previously reported, within each population (CD3+, CD4+ and CD8+ T cells), T cells from three independent biological donors (three biological replicates) exhibited overall similar phenotypic characteristics [7]. Briefly, the surface expression of the early T-cell activation marker CD69 and the middle activation marker CD25 (IL2RA) [22] were rapidly upregulated within 10 hours and 24 hours respectively; T-cell proliferation did not start until 48 hours and cell numbers doubled by 96 hours following T-cell activation. Accordingly, we divided our experimental time course into early T-cell activation (0–10 hours), middle and late T-cell activation (10–48 hours), and T-cell proliferation (48–96 hours). Our microarray results have been validated by Q-RT-PCR assays with a selection of fifteen significant genes covering a broad range of expression patterns and intensities [7]. We have also established the reproducibility of our genome scale transcription data within each population (CD3+, CD4+ and CD8+ T cells) and across the three populations [7]. SAM analysis identified a total of 4167 unique, significant regulated genes in T-cell activation, with similar transcription patterns in three replicate biological experiments within each population [7].

**Figure 1:**
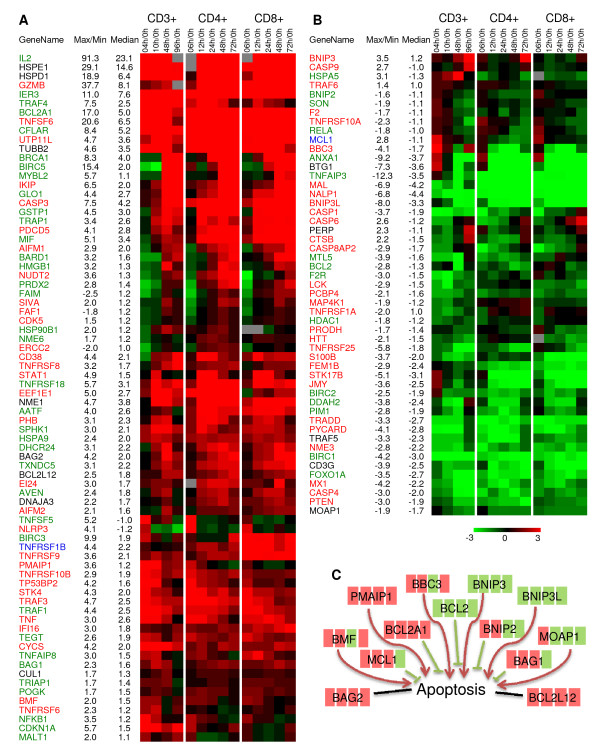
**Expression profiles of genes associated with regulation of apoptosis**. Genes that were differentially expressed temporally in T-cell activation of the three (CD3+, CD4+ and CD8+) populations were divided into two groups (**A **with mostly upregulated genes, and **B **with mostly downregulated genes) according to their distinct expression patterns based on hierarchical clustering using the Euclidean distance metric. Color denotes degree of differential expression compared to 0 hour (saturated red = 3-fold up-regulation, saturated green = 3-fold down-regulation, black = unchanged, gray = no data available). Expression data shown are averages from three independent biological experiments for each T-cell population. The median ratio, along with the maximum (for up-regulated genes) or minimum (for down-regulated genes) ratio of stimulated T cells at each timepoint (with respect to the expression of 0 hour) is provided (a negative value represents down-regulation). Pro-apoptotic genes names and descriptions are shown in red, anti-apoptotic genes are shown in green and genes with both pro- and anti-apoptosis roles are shown in blue. Genes with unknown functions in apoptosis are shown in black. **(C) **Schematic view of significantly regulated genes of BCL2 family. Green and red connections denote negative and positive regulation of apoptosis, respectively, based on information of NCBI website [18]. Regulation of gene transcription in CD3+ T cells, compared to 0 hour, is denoted by different color (green: downregulation, red: upregulation) at each timepoint in the sequence of 4, 10, 48 and 96 hours.

Following SAM analysis, ontological analysis using the MeV EASE module identified 125 significantly regulated genes, associated with 'regulation of apoptosis' (EASE score: 7.65E-08), which shows that there is an active involvement of the apoptotic machinery during T-cell activation and proliferation. Made up of both anti- and pro-apoptotic genes, these 125 genes shared well-preserved expression patterns among the three T-cell populations (Figure 1A and Figure 1B). The mainly upregulated cluster (Figure 1A) contains both anti- and pro-apoptotic genes, and so does the mainly downregulated cluster (Figure 1B). This suggests an essential role of the balance between anti- apoptotic and pro- apoptotic signalling in T-cell activation.

**Figure 2:**
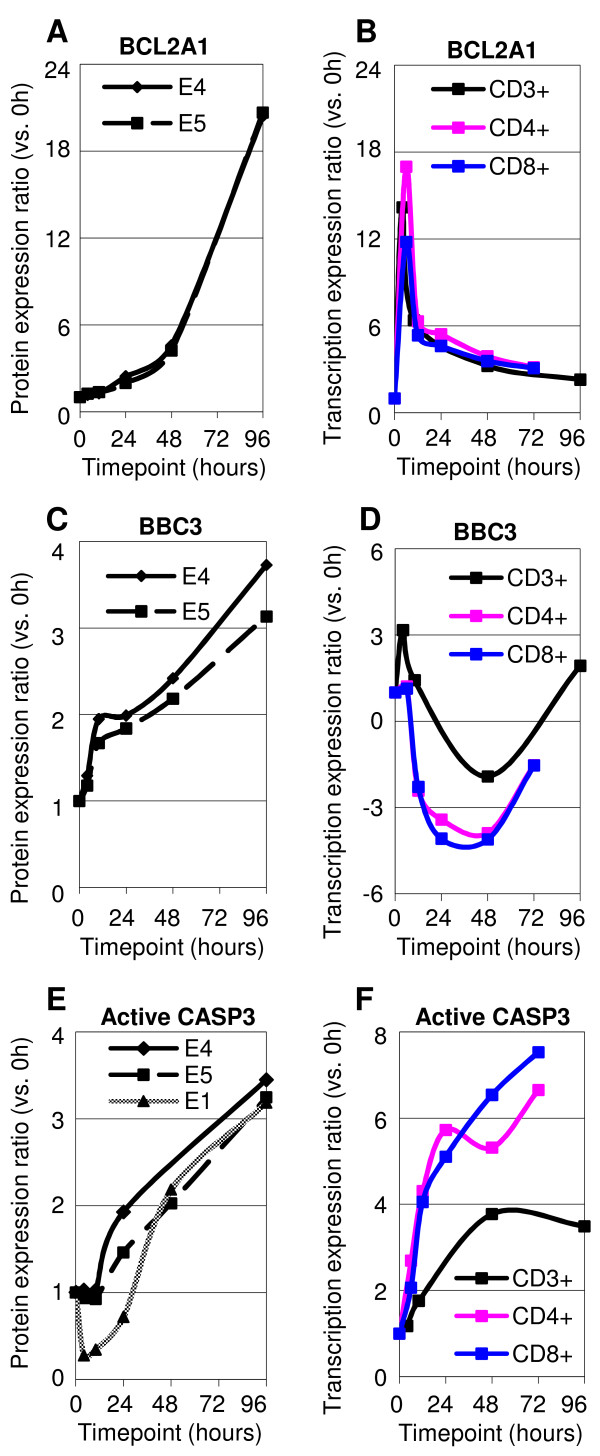
**Protein expression profiles of (A) BCL2A (C) BBC3 and (E) active CASP3**. Protein expression kinetics supports the transcriptional patterns of selected genes demonstrated by microarray analysis. CD3+ T cells were selected, stimulated (with anti-CD3/anti-CD28 antibodies), cultured and harvested at the indicated timepoints of culture to analyze the protein expression by flow cytometric assays. For BCL2A and BBC3, data from two independent experiments, E4 and E5, are shown; for CASP3, data from three independent experiments, E1, E4 and E5, are shown. **(B)**, **(D) **and **(F) **demonstrated the transcriptional patterns of BCL2A1, BBC3 and CASP3 in all three populations (CD3+, CD4+ and CD8+).

Members of the BCL2 family are key regulators of apoptosis. The balance between pro- and anti-apoptotic BCL2 family members determines the cellular fate in response to survival cues and stress signals [23]. The functions and transcriptional regulation of these BCL2 family genes in T-cell activation remain largely unexplored. Alves et al. reported several significantly regulated BCL2 family genes in T-cell activation without discussion [24]. Our microarray data identified a set of significantly regulated BCL2 family genes reported by Alves [24], but with different transcriptional patterns (Figure 1). These included continuously upregulated BCL2A1 (anti-apoptotic), early upregulated MCL1 (myeloid cell leukemia sequence 1) (anti-apoptotic), BMF (BCL2 modifying factor) (pro-apoptotic) and PMAIP1 (pro-apoptotic), late (48 hours) downregulated BCL2 and dynamically (up-down-up) regulated BBC3 (pro-apoptotic). Flow cytometric assays (sample flow cytometry histograms shown in Additional file 2 .)demonstrated the continuous upregulation of BCL2A1 (Figure 2A) and upregulation of BBC3 at 0–10 hours and 24–96 hours at the protein level (Figure 2C), both of which are consistent with their transcriptional patterns. The BCL2A1 gene has been reported as a direct target of transcription factor NF-κB complex, p65/p50, in T cells [25]. This indicates a continuous involvement of BCL2A1 and a constant activity of the NF-κB (p65/p50) complex in T-cell activation. BCL2 has been hypothesized to be able to block T-cell death [26]. The significant regulation of several genes of the BCL2 protein family and their interacting proteins, whose functions and transcription regulation have not been discussed in the context of T-cell activation, calls for attention to their role in T-cell activation. These include upregulated early (anti-apoptotic BNIP2 (BCL2/adenovirus interacting protein 2)), at 10–48 hours (anti-apoptotic BAG1 (BCL2-associated athanogene)), and late BAG2, BCL2L12, and pro-apoptotic BNIP3, and downregulated pro-apoptotic BNIP3L and pro-apoptotic MOAP1 (Figure 1).

Caspases play a central role as executioners in most types of apoptosis, including activation induced cell death (AICD). However they have not been discussed during T-cell activation. Our microarray data show that numerous caspase genes were differentially expressed. Contrary to the signalling mechanism whereby CASP9 becomes activated first, and then in turn activates CASP3 and CASP6 [4], at the transcriptional level, CASP3 was upregulated at 24–96 hours, earlier than CASP9 and CASP6, which were only upregulated at 96 hours. The flow cytometric assay specific for the active form of caspase 3 protein (sample flow cytometry histograms shown in Additional file 2.)  supported the involvement of CASP3 by 48 hours (Figure 2E). CASP8AP2, which is required in CASP8 mediating apoptosis [27], was downregulated at 4–10 hours and then upregulated to the same level of resting T cells at 48–96 hours. CASP1, as well as its adaptor PYCARD [28], and CASP4 displayed decreased expression throughout, suggesting that CASP1 and CASP4 might play important roles in the homeostasis of resting T cells, but not in T-cell activation. Caspase regulatory protein AVEN (reportedly an inhibitor of CASP9 activation) [29] was upregulated at 10–48 hours, concomitantly with the upregulation of CASP9 at 96 hours. CFLAR (CASP8 and FADD-like apoptosis regulator) was significantly and continuously upregulated. CFLAR has different roles in T cells at different stages. It has been reported that CFLAR is induced by restimulaiton in activated T cells, inhibiting FAS-mediated apoptosis [30], while overexpression of CFLAR in naive T cells decreased T-cell proliferation upon anti-CD3/anti-CD28 stimulation [31]. The strong transcriptional upregulation of CFLAR in T-cell activation, not previously reported, suggests an important role during the process of primary T-cell activation.

Some heat shock proteins are involved in apoptosis, but none has been discussed in the context of T-cell activation. HSPE1 and HSPD1 shared continuously elevated expression at 4–96 hours. It has been hypothesized that HSPE1 and HSPD1 form a complex with and facilitate the activation of pro-caspase 3 [32]. Anti-apoptotic interacting heat shock proteins HSPA9 and HSP90B1 [33,34] were also upregulated; their role remains unknown in T-cell activation. Of note, reportedly anti-apoptotic HSPA5 [35], demonstrated distinct transcription patterns in the three populations: it was upregulated in CD3+ T cells especially at 48 hours, but overall downregulated in both CD4+ and CD8+ T cells. HSPA5 has been reported to retain T-cell antigen receptor alpha chain (TCR-alpha) within the endoplasmic reticulum [36]. These different expression patterns among the three T-cell populations indicate that HSPA5 might be involved in the interaction between CD4+ and CD8+ T cells in T-cell activation.

The transcriptional kinetics of other significantly regulated, and not-previously reported in T-cell activation apoptotic genes are also worth discussing briefly. These include members of the inhibitor of apoptosis protein (IAP) family, and genes of programmed cell death (PDCD) proteins. IAPs inhibit apoptosis by interfering with activation of caspase proteins [37]. The differentially expressed genes of the IAP family displayed different expression patterns: BIRC1 and BIRC2 were downregulated, BIRC5 was down-then-upregulated, and BIRC3 was up-down-upregulated. Of note, pro-apoptotic PDCD5 and AIF1 (PDCD8) were upregulated at 10–96 hours.

Non-caspase executor proteases, such as Granzyme B (GZMB) and CTSB (cathepsin B), were also significantly regulated. Besides its function in inducing target cell apoptosis, intracellular degranulated GZMB has also been implicated in AICD in TH2 cells [38]. Our microarray data revealed that GZMB was continuously upregulated with similar transcription patterns in CD4+ T cell and CD8+ T cells, suggesting an involvement of GZMB in T-cell activation. CTSB has been reported to promote T-cell apoptosis by immune-suppressive anti-T cell agents, mitogen antithymocyte globulins (ATGs) [39]. Transcription of CTSB was downregulated first (at 4–48 hours) then upregulated at 96 hours, thus suggesting a role of CTSB in the early stage of T-cell proliferation.

The TNF receptor family plays important roles in extrinsically induced apoptotic pathways. Some TNF receptors have death domains and are directly involved in apoptosis. Transcription of several TNF receptors (TNFRSF6, -8, -9, -18, -1A, -1B, -10A, -10B, and -25) was differentially regulated. Of note, TNFRSF6 (FAS), the well-known AICD receptor, was upregulated at 4–10 hours, but not the other two components of the death-inducing signalling complex (DISC): FADD and CASP8 [40]. FAS has also been implicated in multiple pathways including NF-κB, extracellular signal-regulated protein kinase (ERK) 1 and -2, and p38; however, the function of FAS in early T-cell activation has not been reported [4]. Contrary to the reported induction of death-receptors TNFRSF1A and TNFRSF25 (DR3) in T-cell activation [41,42], our microarray data show that both are downregulated, together with TRADD (TNFRSF1A-associated death domain protein), their common adaptor protein. These data suggest that the apoptosis pathways mediated by TNFRSF1A/TRADD and TNFRSF25/TRADD are suppressed upon T-cell activation. TNFRSF1B was upregulated and more significantly so in CD8+ T cells. The function of TNFRSF1B in T cells remains controversial, with both anti-apoptotic and pro-apoptotic functions reported [43,44]. Furthermore, TNFRSF1B has not been reported to be T-cell subset (CD4+ or CD8+) specific. BIRC3 (also known as IAP1), component of the TNFRSF1B signalling complexes inducing apoptosis [45], was more significantly upregulated in CD8+ T cells, similarly to TNFRSF1B. This suggests that the TNFRSF1B signalling might have CD8+ specific functions. A few TNFSF and TNFRSF related proteins, the function of which remain largely unknown, were significantly regulated. These included FAIM (FAS apoptotic inhibitory molecule), FAF1 (Fas associated factor 1), and SIVA (CD27 (TNFRSF7)-binding protein)), which were downregulated first and then upregulated; and TNFAIP8, which was upregulated first and then downregulated.

Numerous TNF receptors and related proteins involved in NF-κB signalling pathway were significantly regulated in T-cell activation. TNFRSF8, reportedly able to promote apoptosis through inducing the activation of NF-κB complex [46], was significantly upregulated at 48–96 hours. TNFRSF9, reportedly able to promote apoptosis and suppress the activation of NF-κB complex [47], was mainly upregulated. TNFRSF10A (TRAILR1) and TNFRSF10B (TRAILR2), which can interact with several members of TRAFs to activate NF-κB complex [48], together with several members of the TRAF family (TRAF4, TRAF1, TRAF3, and TRAF6) were upregulated. A detailed examination of the transcriptional orchestration of NF-κB signaling pathway in T-cell activation is presented next.

### NF-κB signalling pathway

**Figure 3:**
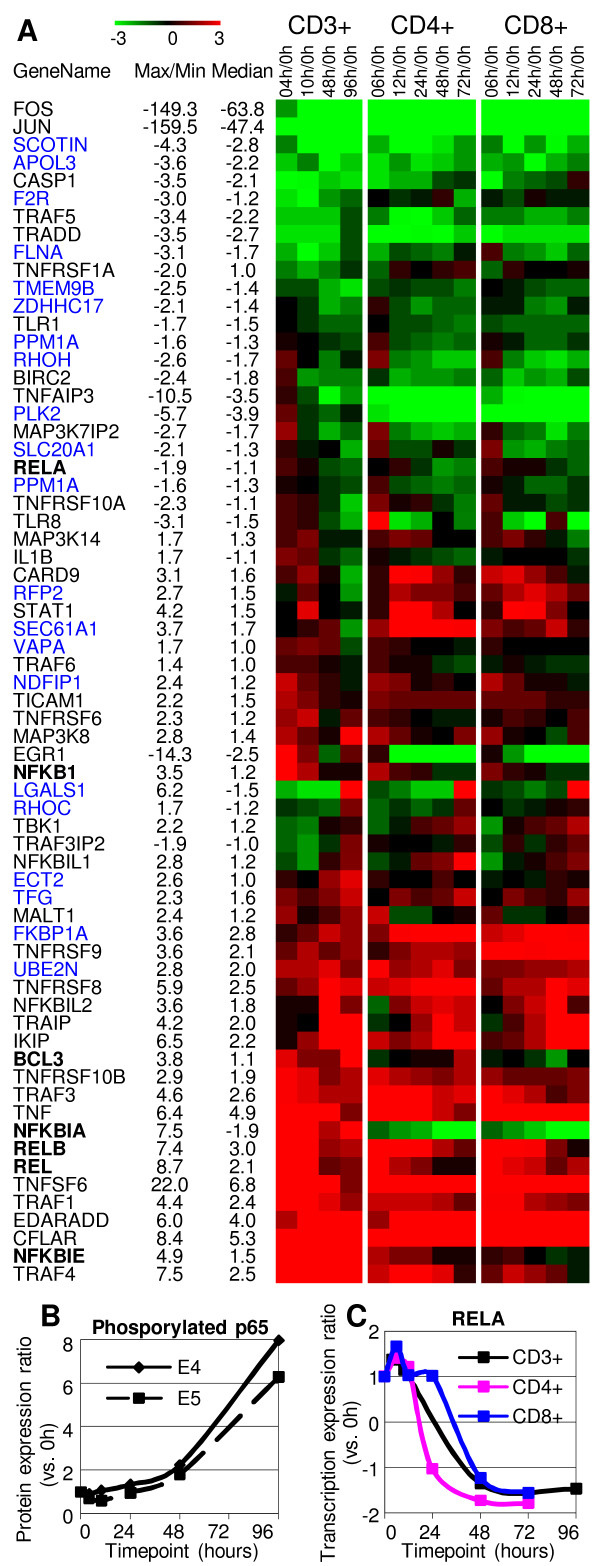
**Significantly regulated genes in NF-κB signalling pathway**. **(A) **Expression profiles of genes involved in NF-κB signalling pathway. Color denotes degree of differential expression compared to 0 hour (saturated red = 3-fold up-regulation, saturated green = 3-fold down-regulation, black = unchanged, gray = no data available). Expression data shown are averages from three independent biological experiments for each T-cell population. The median ratio, along with the maximum (for up-regulated genes) or minimum (for down-regulated genes) ratio of stimulated T cells at each timepoint (with respect to the expression of 0 hour) is provided (a negative value represents down-regulation). Genes, whose transcription can induce the activation of the NF-κB complex, identified in a large scale screening study [60] were shown in blue. **(B) **Protein expression profile of phosphorylated p65 of NF-κB complex. CD3+ T cells were selected, stimulated (with anti-CD3/anti-CD28 antibodies), cultured and harvested at the indicated timepoints of culture to analyze the protein expression by flow cytometric assays. Data from two independent experiments, E4 and E5, are shown. **(C) **The transcriptional pattern of RELA in all three populations (CD3+, CD4+ and CD8+).

**Figure 4:**
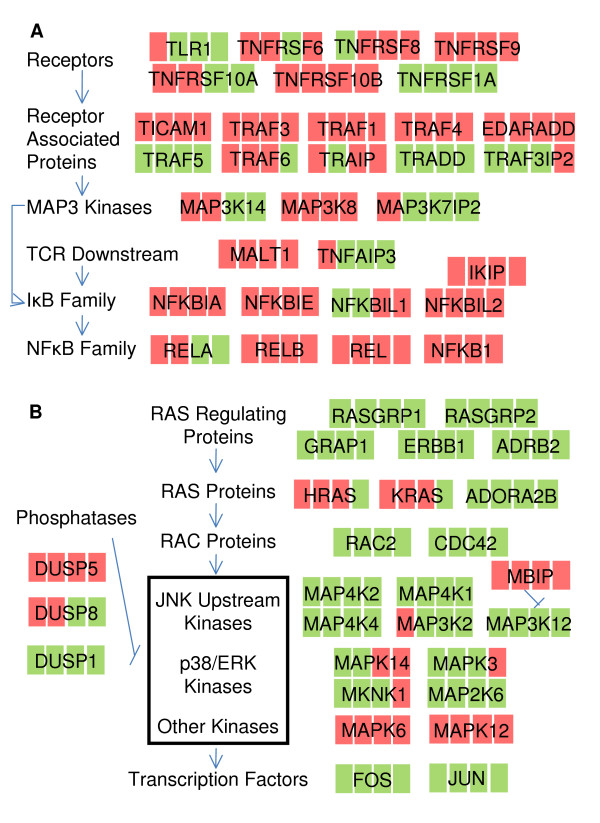
**Pathway schematic of significantly regulated genes in (A) NF-κB signalling and (B) MAP kinase signalling**. Membership was manually determined from the corresponding gene pages in NCBI [18] and references therein. The regulation of gene transcription in CD3+ T cells, compared to 0 hour, is denoted by different color (green: downregulation, red: upregulation) at each timepoint in the sequence of 4, 10, 48 and 96 hours.

The transcription factor NF-κB complex, a collection of several homodimers or heterodimers of Rel proteins (REL, RELA (p65), RELB, p50 and p52), plays a key role for the regulation of T-cell activation by mediating the induction of various genes that control T-cell proliferation, activation and survival [49]. A wide array of stimuli including IL1, and TNF as well as TCR stimulation lead to the onset of cascades that ultimately lead to NF-κB activation [2]. Due to the broad range of the upstream signalings and the complexity of the dimers, our knowledge of the orchestrated regulation of NF-κB signalling pathway and activity of the different NF-κB dimers in T-cell activation is far from complete. The transcriptional regulation of significantly regulated genes associated with the NF-κB signalling pathway is shown in Figure 3A and Figure 4A. NF-κB family genes (REL, RELA (p65), and RELB) and IκB family genes (NFKBIA (the inhibitor of RELA), NFKBIE (inhibitor of REL) and NFKB1 (p105, precursor of p50)) shared similar transcription patterns, an early upregulation at 4–10 hours was followed by a decrease at 48–96 hours. IKIP (IKK interacting protein) was significantly upregulated at 48–96 hours (Figure 3A). We also examined the intracellular protein expression of phosphorylated p65, the major active component of the NF-κB complex. Flow cytometric analysis (sample flow cytometry histograms shown in Additional file 2.) demonstrated an increase of the phosphorylated p65 at 48–96 hours (Figure 3B). This activation delay is likely the result of the strong upregulation of NFKBIA at 4 and 10 hours. It is also possible that p65 might quickly become activated within 4 hours leading to the transcriptional induction of NFKBIA, one of the target genes of the NF-κB complex [50]. Of note, not only the transcriptional regulation of RELB (maximum fold change of 7.4) was more significant than that of RELA (maximum fold change of 6.5), but also RELB had higher transcriptional levels than RELA. BCL3, a transcriptional coactivator of NF-κB homodimer p50/p50 and p52/p52 [51], was upregulated at 4 and 96 hours.

TLR1 (toll-like receptor) and TICAM1 (toll-like receptor adaptor), which promote activation of the NF-κB complex [52], showed decreased expression at 48–96 hours, suggesting that this upstream activation cascade of the NF-κB complex might not be active during early T-cell proliferation. Also involved in NF-κB complex activity regulation, members of the TNF receptor super family (TNFRSF6, -8, -9, -10A, and -10B), their associated proteins (TRAF1, -3, -4 and -6, EDARADD (ectodysplasin A receptor-associated)) and TRAF interacting protein (TRAIP) showed increased expression. TNFRSF-10A, -10B, TRAF1, -3, and -6 were upregulated at 4–10 hours and TRAIP, which inhibits the TRAF-mediated NF-κB activation [53], was upregulated at 48–96 hours. This orchestrated gene expression regulation suggests that the TRAF-mediated upstream signalling of NF-κB activation is active at 4–10 hours. Furthermore, the early upregulation of MAP3 Kinases (MAP3K14, MAP3K8, MAP3K7IP2) supports the early involvement of the TNF receptor pathway since MAP3 Kinases activate IκB kinases recruited by TRAFs to the TNF receptor complex [54]. In contrast, TNFRSF1A, TRADD (TNFRSF1A-associated via death domain) and TRAF5 were downregulated, suggesting that the reported TNFRSF1A-TRADD-TRAF2 cascade [55] and TRAF5 mediated cascade [56], regulating the activation of NF-κB complex, might not be active in T-cell activation.

TCR specific signalling protein MALT1 (through the CARD11-BCL10-MALT1 complex) was recently reported to be required for optimal NF-κB activation through proteolysis of the NF-κB inhibitor TNFAIP3 [57,58]. However the transcriptional regulation of MALT1 and TNFAIP3 in T-cell activation has not been reported. Our microarray data demonstrated that MALT1 was upregulated at 4 and 96 hours, and TNFAIP3 was upregulated at 4 hours and downregulated at 10–96 hours. CARD11 and BCL10 were not identified as significantly regulated, however. CARD9, the equivalent gene of CARD11 in dendritic cells [59], was upregulated at 4 and 10 hours, suggesting its involvement in T-cell activation.

Matsuda et al. identified genes whose transcription can induce the activation of the NF-κB complex by introducing cDNA clones of full-length human cDNA libraries to HEK 293 cells [60]. Our microarray data demonstrated that several of these genes were significantly regulated with different transcription patterns (gene names shown in blue in Figure 3A). Some of these genes were upregulated, such as UBE2N, ECT2, TFG, and FKBP1A; while others were downregulated, such as SCOTIN, APOL3 and FLNA. Two NF-κB inhibitor-like proteins (NFKBIL1 and NFKBIL2), whose function has not been determined, were significantly upregulated at 96 hours and 48 hours respectively, suggesting that they are involved in early T-cell proliferation and late T-cell activation, respectively.

Some genes (EGR1, NFKBIA and NFKBIE) had significantly different expression patterns in CD4+ and CD8+ T cells compared to CD3+ T cells. NFKBIA and NFKBIE are inhibitors of REL proteins, and EGR1 has been reported to inhibit the activity of RELA [61]. It is possible that the communication between CD4+ and CD8+ T cells may affect the activation of the NF-κB complex. Activation of the NF-κB complex is further supported by the significant regulation of many target genes, whose transcription is regulated by NF-κB complex upon anti-CD3/anti-CD28 stimulation (Additional file 1). Interestingly, some of these target genes showed higher expression in resting T cells.

### MAP kinase signalling

**Figure 5:**
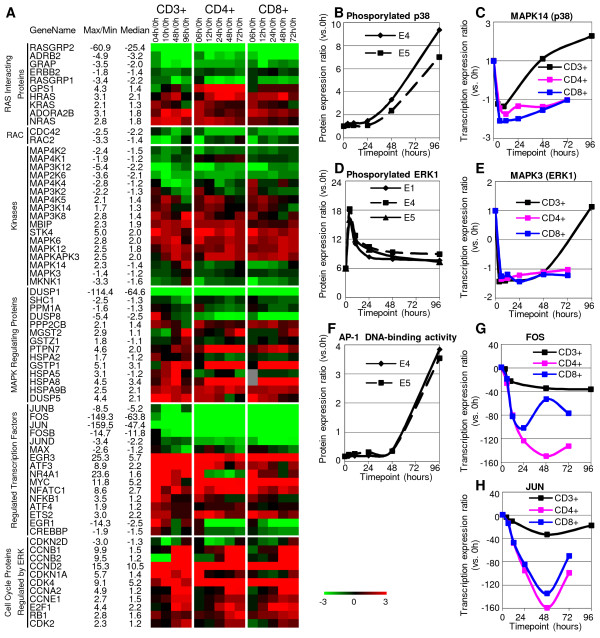
**Significantly regulated genes involved in MAP kinase signalling**. **(A) **Expression profiles of genes that belong to the list of MAP kinase pathways. Color denotes degree of differential expression compared to 0 hour (saturated red = 3-fold up-regulation, saturated green = 3-fold down-regulation, black = unchanged, gray = no data available). Expression data shown are averages from three independent biological experiments for each T-cell population. The median ratio, along with the maximum (for up-regulated genes) or minimum (for down-regulated genes) ratio of stimulated T cells at each timepoint (with respect to the expression of 0 hour) is provided (a negative value represents down-regulation). **(B) **Protein expression profiles of phosphorylated p38 and of **(D) **phosphorylated ERK1 agree with the late transcription upregulation of MAPK8 and MAPK3. CD3+ T cells were selected, stimulated (with anti-CD3/anti-CD28 antibodies), cultured and harvested at the indicated timepoints of culture to analyse the protein expression by flow cytometric assays. Data from two independent experiments, E4 and E5, are shown. **(F) **DNA-binding activity profile of transcription factor AP-1 captured the immediate and transient activation of AP-1 in T-cell activation. CD3+ T cells were selected, stimulated (with anti-CD3/anti-CD28 antibodies), cultured and harvested at the indicated timepoints of culture. Data from three independent experiments, E1, E4 and E5, are shown. **(C)**, **(E), (G) **and **(H) **demonstrated the transcriptional patterns of MAPK14 (p38), MAPK3 (ERK1), FOS and JUN (the two major components if AP-1) in all three populations (CD3+, CD4+ and CD8+).

Mitogen-activated protein (MAP) kinases are important signalling mediators in regulation of apoptosis, including the anti-apoptotic ERKs, the anti-/pro-apoptotic c-Jun N-terminal kinases (JNKs), and anti-/pro-apoptotic p38-MAPKs. Yet the mechanisms as to how these MAP kinases regulate apoptosis remain controversial [62]. It has been suggested that the three main mammalian cell MAP kinase cascades, JNK, p38 and ERK, are essential for T-cell functions [63,64]. However, the temporal regulation of these MAP kinase cascades in T-cell activation remains largely unexplored. Thus, we focused on the significantly regulated genes involved in MAP kinase signalling pathway (Figure 4B and Figure 5). A few genes of the RAS family and RAS regulating protein were upregulated upon anti-CD3/anti-CD28 T-cell stimulation, including HRAS (at 10–48 hours), KRAS (at 4 hours), NRAS (at 4–96 hours) and ADORA2B (at 4–96 hours). HRAS, KRAS and NRAS have been reported to be activated shortly after TCR stimulation [65], however the regulation of their expression (either at the transcriptional or protein level) has not been reported. ADORA2B reportedly regulates ERK and p38 MAP kinase cascades in mast cells [66], but its function in T cells is not known. Contrary to upregulated RAS genes, several upstream RAS regulating proteins (RASGRP1, -2, ADRB2, GRAP, ERBB2) showed higher expression in resting T cells (Figure 5A). The downregulation of RASGRP1 and GRAP does not correlate with their reportedly positive roles in T-cell receptor signalling [67,68]. Little is known about RASGRP2, ADRB2, and ERBB2 in the context of T-cell activation. Contrary to the reported activation of RAC proteins CDC42 and RAC2 [69,70] in T-cell activation, here we found that their transcription was downregulated.

Several kinases in the MAP kinase signalling pathway were differentially expressed. A few kinases upstream of the JNK cascade (MAP4K2 [71], MAP4K1 [72], MAP3K12 [73], MAP4K4 [74], MAP3K2 [75]) were downregulated, and MBIP (MAP3K12 binding inhibitory protein), which inhibits the MAP3K12 mediated JNK activation [76], was upregulated, suggesting that the JNK cascade might not be active at 4–96 hours. MAPK14 (p38), MAPK3 (ERK1), MKNK1 (the interacting protein of both p38 and ERK1 [77]) and MAP2K6 (p38 specific MAP kinase kinase [78]) displayed similar regulation patterns: downregulated at 4–10 hours and then upregulated at 48–96 hours. The flow cytometric assays (sample flow cytometry histograms shown in Additional file 2.) of phosphorylated p38 and ERK1 confirmed their transcriptional patterns, namely that there was no significant increase of phosphorylated p38 until 24 hours and phosphorylated ERK1 until 48 hours, but large increases after that until 96 hours (Figure 5B and Figure 5D). Kinases involved in positive regulation of activity of the NF-κB complex, MAP3K14 [79] and MAP3K8 [80], showed increased expression at 4–10 hours which is consistent with expression patterns of several members of NF-κB and IκB family genes (Figure 3A). Two less examined kinases, MAPK6 and MAPK12, were mainly upregulated at 4–48 hours and at 10–96 hours respectively, suggesting that they are actively involved in T-cell activation.

Several MAP kinase regulating proteins were also differentially expressed upon anti-CD3/anti-CD28 activation of T cells. Members of the dual specificity phosphatase family, negatively regulating members of the MAP kinase family, show different expression patterns. DUSP1, able to inactivate ERK1, JNK and p38 [81], was significantly downregulated throughout. In contrast, DUSP5, specific inhibitor of ERK1 [82], was strongly upregulated at 4–10 hours and decreased thereafter in concert with the upregulation of ERK1 at 48–96 hours at both the transcriptional and protein level (Figure 5A and Figure 5C). DUSP8, of which little is known, was significantly upregulated at 4 hours and then downregulated at 48–96 hours, which is opposite to the transcriptional pattern of MAPK14 and MAPK3. Some transcription factors regulated by MAP kinase pathway were downregulated, and most intensely so were FOS and JUN. FOS and JUN proteins are the main components of the transcription factor complex AP-1. AP-1 has been reported to be quickly activated in response to T-cell activation [83], which is contradictory to the significant downregulation of FOS and JUN. The temporal activity of AP-1 in T-cell activation is not known. Thus, we examined the DNA-binding activity of AP-1, which rapidly increased within 4 hours and then rapidly decreased (Figure 5D). It is possible that FOS and JUN were immediately and transiently regulated upon anti-CD3/anti-CD8 stimulation, and that their upregulation was not captured by our first timepoint following T-cell activation.

## Discussion

In this work, we sought to improve our understanding of regulation of apoptosis in T-cell activation. We approached this problem by analysing global, temporal microarray data from ex vivo CD3+, CD4+ and CD8+ T-cell cultures, and leveraging Gene Ontology associations and prior knowledge. This approach extended our knowledge base in three ways: we presented detailed kinetic gene expression information on genes with previously hypothesized or presumed important roles in apoptosis; we identified a new set of genes not previously associated with T-cell activation; and, we integrated and connected the previous knowledge with temporal transcription profiles to build a more comprehensive picture of regulation of apoptosis in T-cell activation. Gene expression data were further explored by examining protein expression (active form/phosphorylated form) and functional activity levels as a first assessment of their functional role.

Genome-scale transcription profiling provides the opportunity to more holistically evaluate the regulation of a group of genes, of the same family, or with analogous functions, or associated in specific pathways. Composed of several members, the BCL2 family proteins are key players in regulation of apoptosis. BCL2, BAX, BAK have been reported to play important roles in apoptosis post activation in T cells [4]. Knockout of pro-apoptotic BBC3 and PMAIP1 decreased DNA damage-induced apoptosis in mice fibroblasts, but only loss of BBC3 protected lymphocytes from cell death [84]. However, the function and transcriptional regulation of most of the BCL2 family members and their regulatory proteins remain unexplored in T-cell activation. Our data suggest the distinct stages that BCL2 family members are involved in the process of T-cell activation, thus providing directions for future studies. For instance, validated by protein abundance assays (Figure 2A and 2B), the functions of continuously upregulated BCL2A1 and dynamically regulated BBC3 deserve further studies in T-cell activation. The synchronized early transcriptional upregulation of MCL1 and its inhibitory interacting proteins PMAIP1 [24] and BMF [85] imply their involvement in T-cell activation quickly upon TCR ligation.

In typical apoptosis signalling, CASP9 is activated first, which leads to activation of downstream effectors CASP3, and CASP6. Here, CASP3 was upregulated (at both the transcriptional and protein levels) by 48 hours, compared to the upregulation of CASP9 only at 96 hours. These findings suggest that active CASP3 protein might have a different role in T-cell activation and thus its activity might be regulated by other proteins, such as HSPE1 and HSPD1 (Figure 1A), rather than CASP9. Surprisingly, the well-known AICD receptor, TNFRSF6 (FAS), was upregulated at 4–10 hours, suggesting the involvement of FAS immediately upon T-cell activation.

Our data show that numerous significantly regulated genes associated with apoptosis are involved in NF-κB signalling pathway and MAP kinase signalling pathway. Supported by the increase of phosphorylated p65 at 48–96 hours (Figure 3B), the simultaneous upregulation of NF-κB family genes (REL, RELA, and RELB) and IκB family genes (NFKBIA, NFKBIE and NFKB1) at 48–96 hours (Figure 3A) suggests that multiple versions of the NF-κB dimmer complex are active during this time period in our experiments. Lack of early detection of phosphorylated p65 that multiple versions of the NF-κB dimmer complex are active during this time period in our experiments could be the result of the strong upregulation of NFKBIA at 4–10 hours. It is also possible that p65 might immediately and transiently become activated, and then quickly deactivated within 4 hours, our first timepoint. Of note, IKIP (IκB kinase interacting protein) was significantly upregulated at 48–96 hours (Figure 3A). To date, the function of IKIP remains unknown. Its transcriptional kinetics suggests that it might have a positive role in NF-κB activity regulation.

Validated by the increase of phosphorylated p38 and ERK1 at 24–96 hours and 48–96 hours, respectively, as measured by flow cytometry assays (Figure 5B and Figure 5C), the similar transcriptional patterns of MAPK14 (p38), MAPK3 (ERK1), MKNK1 (the interacting protein of both MAPK14 and MAPK3 [77]) and MAP2K6 (p38 specific MAP kinase kinase [78]) (downregulated at 4–10 hours and then upregulated at 48–96 hours) suggest that cascades of p38 and ERK1, but not JNK, are synergistically activated during late T-cell activation and early proliferation. Activity of transcription factor AP-1, mostly regulated by JNK and p38 cascades [86], increased immediately, but only transiently, upon anti-CD3/anti-CD28 stimulation, suggesting a potentially rapid but transient activation of JNK and p38 cascades in T-cell activation. Significantly, there was no activity increase of AP-1 at 48–96 hours, in contrast to the increase of phosphorylated p65. It has been suggested that MAP kinases have different roles in CD4+ and CD8+ T cells [87]. However most of the significant regulated genes in MAP kinase signalling pathway shared similar expression patterns between the CD4+ and CD8+ subsets. It is possible that the MAP kinase signalling pathway is regulated similarly between the two subsets in the context of T-cell activation. It is also possible that the MAP kinases might function differently due to regulation at protein level.

T-cell activation using bead-conjugated-anti-CD3/anti-CD28 antibodies is an established state-of-the art method [88] for both fundamental studies, such as gene-expression profiling [6,89], as well as for clinical immunotherapy applications [90]. It provides both the stimulatory and costimulatory signals [91,92] and the physical contact, in place of APCs [93], and as such it is assumed to mimic the in vivo situation. Consistent with that assumption, and for well-established genes partaking in the T-cell activation process, our data are consistent with the large body of literature (a good fraction of which comes from in vivo studies) on T-cell activation.

## Conclusion

Our study captured novel temporal patterns of previously known but many novel, in the context of T-cell activation, genes ontologically classified under the term 'regulation of apoptosis'. These patterns were reproducibly and robustly identified as donor independent. Comprehensively integrating previous knowledge, we identified novel significant genes associated with regulation of apoptosis in T cells, as well as with NF-κB and MAPK signalling pathways. This study improves our understanding of the biology and the underlying regulation mechanisms of apoptosis in T-cell activation in the natural CD3+ population, as well as in the CD4+ and CD8+ subsets.

## Competing interests

The authors declare that they have no competing interests.

## Authors' contributions

MW and DW carried out the culture experiments and performed the microarray experiments. MW analysed the microarray data and wrote the manuscript with assistance from EP. MW and EP conceived, designed, and coordinated the study.

## Pre-publication history

The pre-publication history for this paper can be accessed here:



## Supplementary Material

Additional file 1 **Expression profile of NF-κB target genes in T-cell activation of the three (CD3+, CD4+ and CD8+) populations.** Hierarchical clustering (Euclidean distance metric) shows the temporal expression. Color denotes degree of differential expression compared to 0 hour (saturated red = 3-fold up-regulation, saturated green = 3-fold down-regulation, black = unchanged, gray = no data available). Expression data shown are averages from three independent biological experiments for each T-cell population. The median ratio, along with the maximum (for up-regulated genes) or minimum (for down-regulated genes) ratio of stimulated T cells at each timepoint (with respect to the expression of 0 hour) is provided (a negative value represents down-regulation). Click here for file

Additional file 2 **Flow cytometry histograms, at 0 hour and 96 hours in CD3+ population.** Representative (CD3+ experiment, E4) flow cytometry histograms, at 0 hour and 96 hours, of BCL2A1, BBC3, active CASP3, phosphorylated p65, phosphorylated p38 and phosphorylated ERK1. Click here for file
