# Supplementary figures and images for: A global transcriptional view of apoptosis in human T-cell activation
Source: BMC Med Genomics. 2008 Oct 23;1:53. doi: 10.1186/1755-8794-1-53 (PMC2600644; doi:10.1186/1755-8794-1-53)

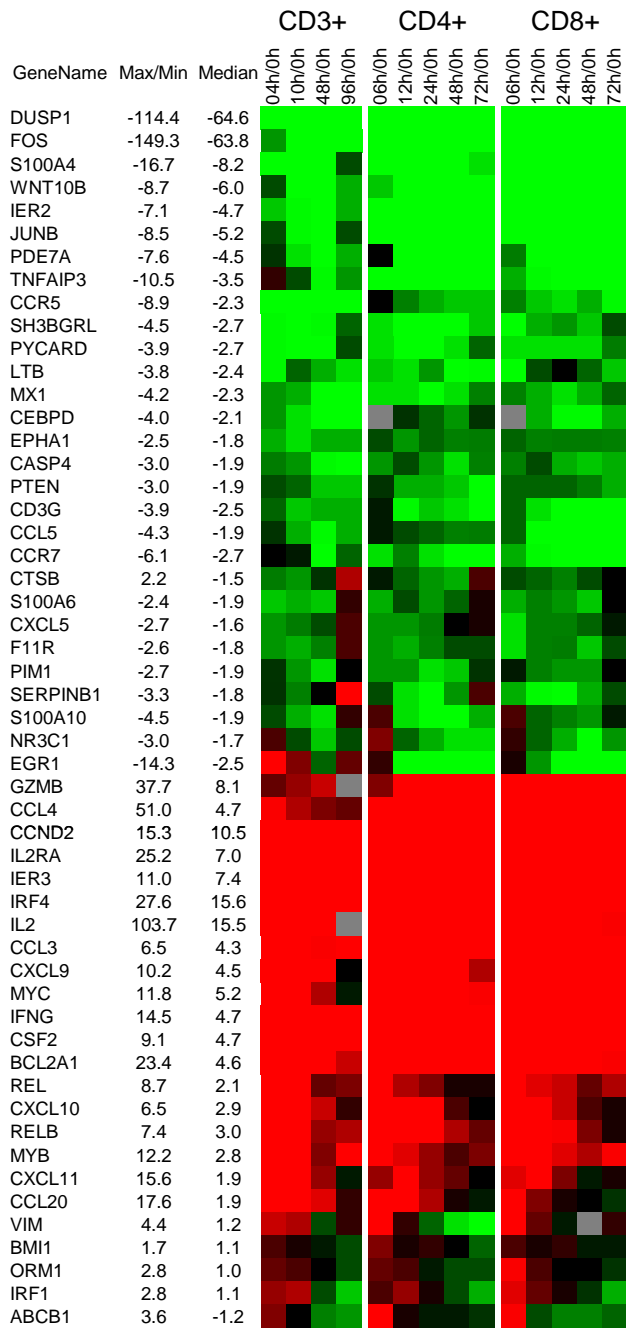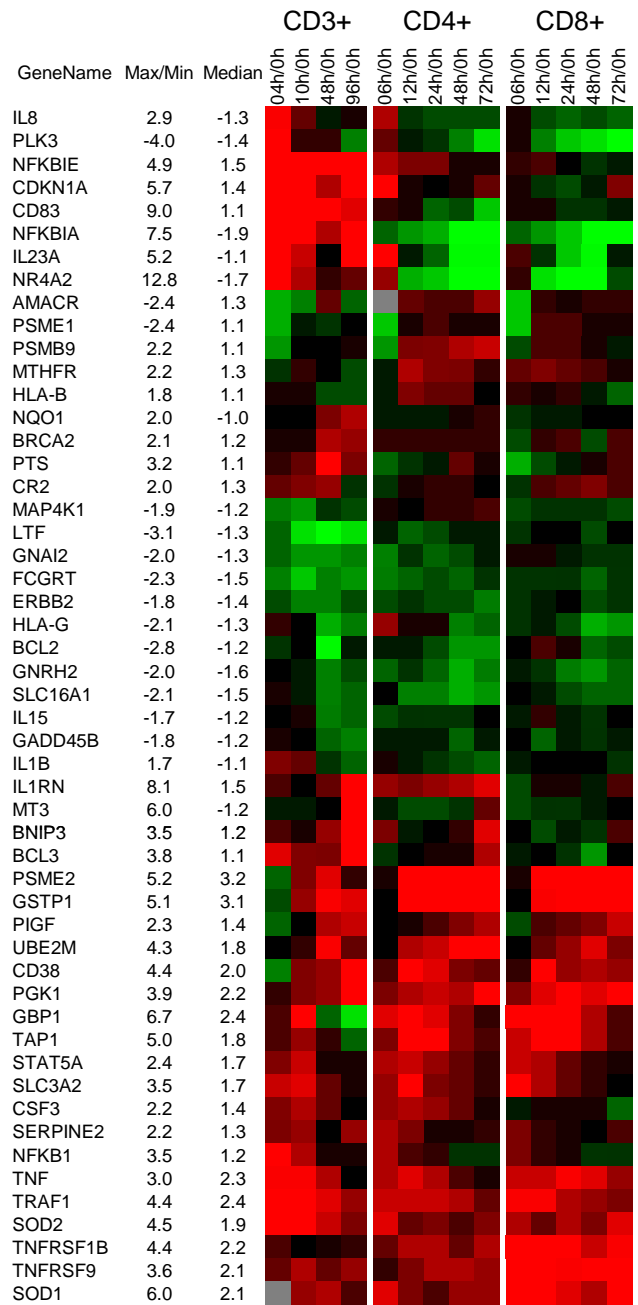

-3 0 3

Supplement: Additional file 1 — Expression profile of NF-κB target genes in T-cell activation of the three (CD3+, CD4+ and CD8+) populations. Hierarchical clustering (Euclidean distance metric) shows the temporal expression. Color denotes degree of differential expression compared to 0 hour (saturated red = 3-fold up-regulation, saturated green = 3-fold down-regulation, black = unchanged, gray = no data available). Expression data shown are averages from three independent biological experiments for each T-cell population. The median ratio, along with the maximum (for up-regulated genes) or minimum (for down-regulated genes) ratio of stimulated T cells at each timepoint (with respect to the expression of 0 hour) is provided (a negative value represents down-regulation). [file 1755-8794-1-53-S1.pdf]

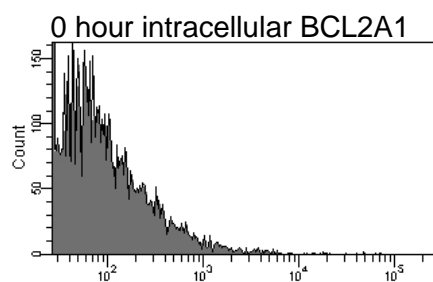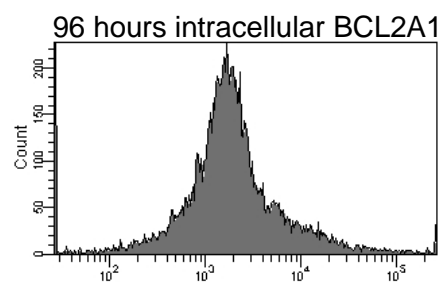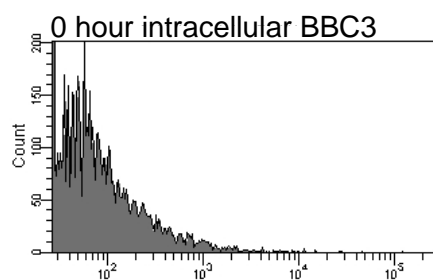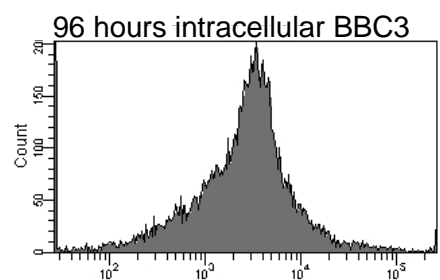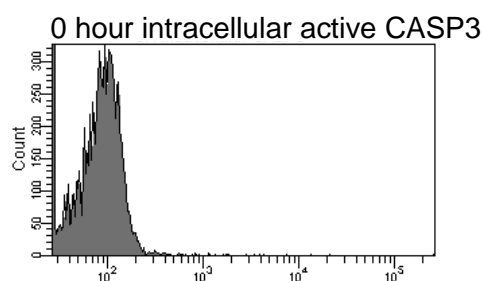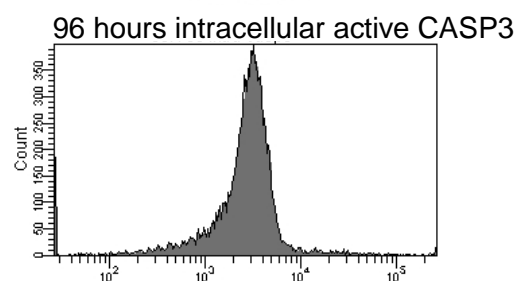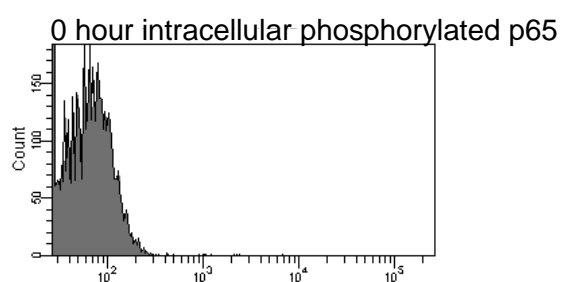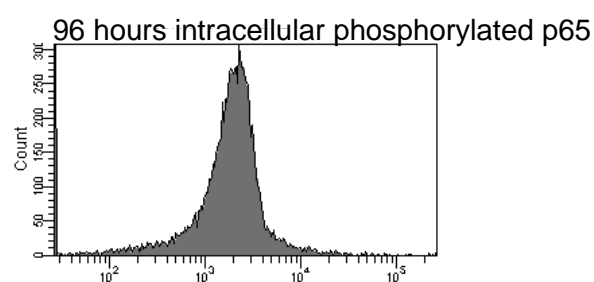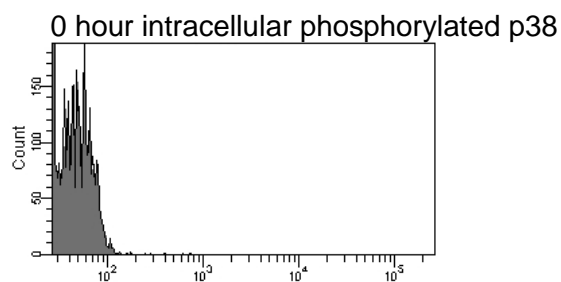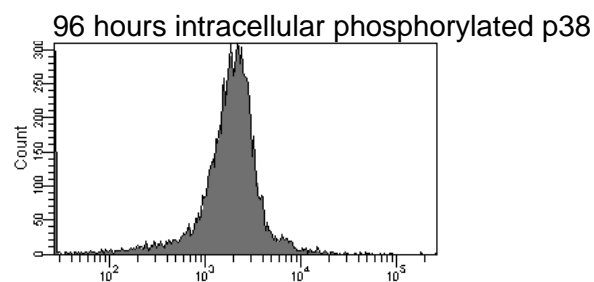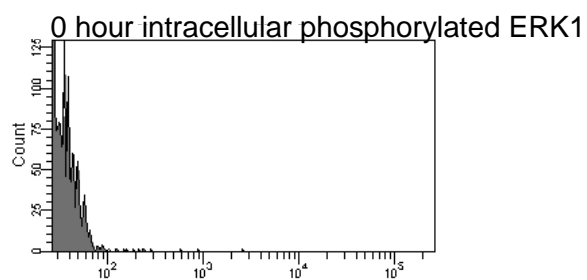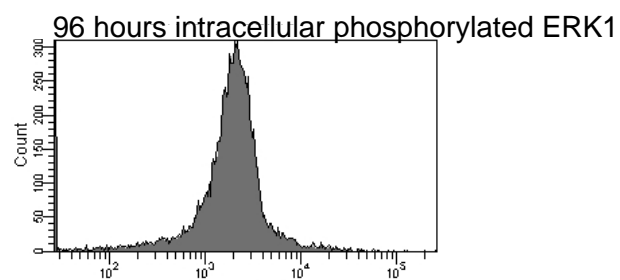

Supplement: Additional file 2 — Flow cytometry histograms, at 0 hour and 96 hours in CD3+ population. Representative (CD3+ experiment, E4) flow cytometry histograms, at 0 hour and 96 hours, of BCL2A1, BBC3, active CASP3, phosphorylated p65, phosphorylated p38 and phosphorylated ERK1. [file 1755-8794-1-53-S2.pdf]
